# “Daily life of CAPS patients treated with canakinumab (Ilaris^®^) : data from the French observational study - ENVOL Study”

**DOI:** 10.1186/1546-0096-13-S1-P162

**Published:** 2015-09-28

**Authors:** I Koné-Paut, P Quartier, O Fain, G Grateau, P Pillet, P Le Blay, F Bonnet, V Despert, K Stankovic, L Willemins, S Queré, O Reigneau, E Hachulla

**Affiliations:** 1 CHU de Bicêtre, CEREMAI, pediatric rheumatology, Le Kremlin Bicêtre, France; 2 Necker Hospital, Pediatric Immunology and Rheumatology, Paris, France; 3 Saint Antoine Hospital, Internal Medicine, Paris, France; 4 Tenon Hospital, Internal Medicine, Paris, France; 5 CHU de Bordeaux, Pediatric Rheumatology, Bordeaux, France; 6 CHU Arnaud de villeneuve, Rheumatology, Montpellier, France; 7 Hôpital Saint André, Internal medicine, Bordeaux, France; 8 CHU de Rennes, Pediatrics, Rennes, France; 9 Novartis pharma, Rueil-Malmaison, France; 10 CHU de Lille, Internal Medicine, Lille, France

## Background

The long-term efficacy of canakinumab has not been thoroughly evaluated since its first use in France in 2007 and its approval for CAPS in 2010. In addition, changes in the daily lives of patients (and their caregivers) since they have received this drug have never been reported.

## Objectives

A multicentre retrospective, observational study set up to assess the “real life” use of canakinumab in all French CAPS patients ever treated since 2007, to describe their clinical course on a long-term, and to analyse changes in their (and their caregiver's) quality of life.

## Methods

We targeted the 70-80 patients ever treated for CAPS in France, at least once and even during a clinical trial. Patients' (parents end caregivers) gave their informed consent to enter the study. Investigators were known experts in the field of CAPS in France who accepted to take part in the study. Data were collected through questionnaires by phone interviews and medical chart reviews, at treatment initiation, 6 months, 12 months and at the last medical visit. They included: clinical data, canakinumab use in real life conditions, impact on patients' (and caregivers') quality of life, and care consumption. The significance limit was set at 5% for all of the statistical tests.

## Results

68 CAPS patients, >90% of the target number, were enrolled (23 children, 45 adults). Sixteen patients (24%) had FCAS, 43 (63%) had MWS and 9 (13%) had NOMID-CINCA. The median duration of treatment was 5 years (from July 2007 to July 2014). >95% of patients remained on treatment. Doses were not modified in nearly half cases (31/68). For 37 patients, dosage adjustments (more often increase) were required (102 in total), especially in younger patients and those with the most severe phenotypes. All clinical symptoms monitored during the study got better under canakinumab. The global activity of the disease, the skin disorders and most of the symptoms were significantly better (p<0.001) at the different study timelines. The evolution of the quality of life score showed a significant improvement (median was at 8 before canakinumab versus 2 after; p<0.0001). Canakinumab treatment allowed also improvement in patient's daily activities, mood, and social life. Patients reported less school absences (79% versus 36%), and less sick leaves (48% versus 6%) after the initiation of canakinumab. The effect was weaker in heavily handicapped CINCA patients; due in part to the late initiation of anti IL1 treatment. Caregivers (49) were mostly family members and 35% of them had CAPS. They dedicated a mean of 7 hours/week to the CAPS patients before treatment and 4 hours during the last year. They spent on average 11.1 days per year of their job before canakinumab treatment versus 3 days after. The trend was less pronounced for caregivers of NOMID-CINCA patients.

## Conclusion

Even retrospectively fashioned, the ENVOL study showed real-life results similar to those obtained during the phase III clinical trials with canakinumab, reinforcing its sustained activity. The maintenance of more than 95% of patients on therapy confirmed its major benefit to CAPS patients, which was demonstrated herein by the positive impact of canakinumab in patients (and their caregivers) social, emotional, educational and professional lives.

